# Assessing Phototoxicity in a Mammalian Cell Line: How Low Levels of Blue Light Affect Motility in PC3 Cells

**DOI:** 10.3389/fcell.2021.738786

**Published:** 2021-12-17

**Authors:** Rana A. Alghamdi, Marino Exposito-Rodriguez, Philip M. Mullineaux, Greg N. Brooke, Philippe P. Laissue

**Affiliations:** ^1^ Department of Chemistry, Science and Arts College, Rabigh Campus, King Abdulaziz University, Jeddah, Saudi Arabia; ^2^ School of Life Sciences, University of Essex, Colchester, United Kingdom; ^3^ Sainsbury Laboratory, University of Cambridge, Cambridge, United Kingdom

**Keywords:** fluorescence, microscopy—light, live imaging, reactive oxygen species (ROS), light intensity (irradiance)

## Abstract

Phototoxicity is a significant constraint for live cell fluorescence microscopy. Excessive excitation light intensities change the homeostasis of the observed cells. Erroneous and misleading conclusions may be the problematic consequence of observing such light-induced pathophysiology. In this study, we assess the effect of blue light, as commonly used for GFP and YFP excitation, on a motile mammalian cell line. Tracking PC3 cells at different light doses and intensities, we show how motility can be used to reliably assess subtle positive and negative effects of illumination. We further show that the effects are a factor of intensity rather than light dose. Mitotic delay was not a sensitive indicator of phototoxicity. For early detection of the effect of blue light, we analysed the expression of genes involved in oxidative stress. This study addresses the need for relatively simple and sensitive methods to establish a dose-response curve for phototoxicity in mammalian cell line models. We conclude with a working model for phototoxicity and recommendations for its assessment.

## Introduction

### Phototoxicity in Live Fluorescence Microscopy

In the context of live fluorescence microscopy, phototoxicity describes the phenomenon by which the light used for fluorescence excitation leads to physiological changes in the observed living sample, be that single cells in culture or a multicellular organism such as a zebrafish. With the excitation light intensities widely used in fluorescence microscopy, these physiological changes are often severe and detrimental and may lead to significant alterations in the biochemistry, physiology and dynamic behaviour of the observed sample. It is also possible that, when observing physiological processes for the first time, more subtle phototoxic effects may go unnoticed as the unperturbed activity is unknown. In either case, the conclusions drawn from these observations could be erroneous and, more dangerously, misleading—since we are not observing a living sample in homeostasis, but documenting the light-induced pathophysiological changes caused by the microscopy method.

### Reactive Oxygen Species in Phototoxicity

**FIGURE 1:**
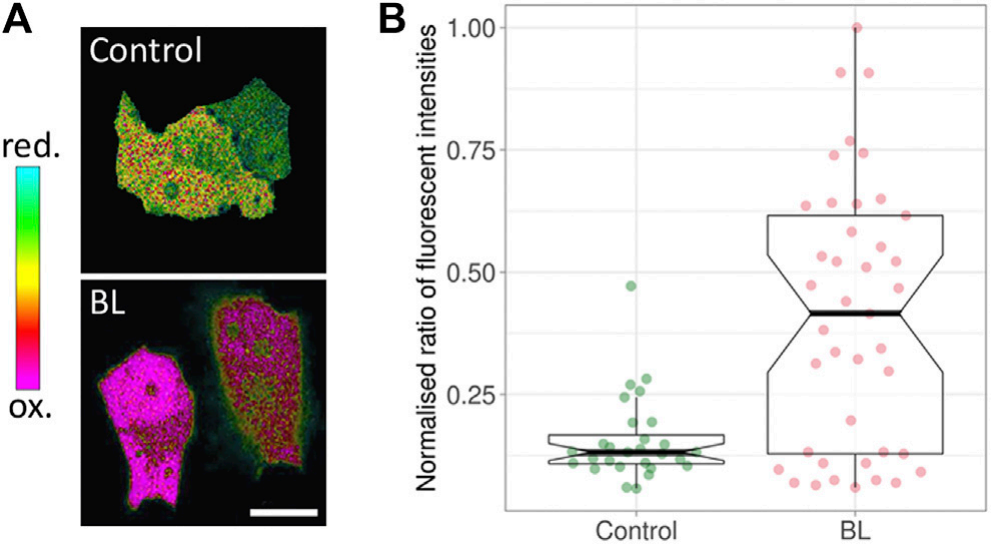
Response of HyPer, a ratiometric hydrogen peroxide biosensor. **(A)** Green Monkey Kidney cells containing HyPer. The control cells (top) were not exposed to blue light prior to taking the ratiometric image, while the cells at the bottom were imaged immediately after being exposed to 77 mW/cm^2^ for 5 minutes of blue light (BL). The noted oxidation state indicates the presence of H_2_O_2_. **(B)** The corresponding box plots. BL: blue light. The middle line in the box represents the median value. Modified from (Alghamdi, 2017).

A key factor of phototoxicity is the generation of free radicals—reactive chemical species with a single unpaired electron in an outer orbit (Riley, 1994; Greenbaum et al., 2000; Dröge, 2002; Redmond and Kochevar, 2006). This unstable configuration promotes reactions with adjacent molecules such as lipids, carbohydrates, and nucleic acids. The majority of free radicals relevant to photodamage are reactive oxygen species (ROS) (Diaspro et al., 2006; Laloi and Havaux, 2015; Kiepas et al., 2020). Several ROS species exist, such as superoxide, hydroxyl and hydrogen peroxide. To demonstrate the generation of hydrogen peroxide upon blue light illumination (Figure 1), a mammalian cell line was transfected with HyPer, a ratiometric biosensor for hydrogen peroxide (Belousov et al., 2006; Markvicheva et al., 2011). The ratio changed immediately after illuminating at a low intensity of 77 mW/cm^2^ for 5 minutes, with the HyPer signal increasing towards the oxidative state (Figure 1; Alghamdi, 2017). The generation of ROS using blue light has been shown in other cell types (Seko et al., 2001; Dixit and Cyr, 2003; Becker et al., 2016; Icha et al., 2017; Yuan et al., 2017).

ROS are important signalling molecules and play key roles in many physiological and pathological processes: stress response, apoptosis, activation of signal cascades, gene expression changes, normal development and regulation of lifespan (Cadenas and Davies, 2000; Hancock et al., 2001; Poli et al., 2004). Intensity and duration of the exposure to ROS determines their effect on a given cell. ROS are a product of normal cellular functioning, but excessive amounts can cause deleterious effects. While low concentrations of ROS can be buffered by the cell without damaging it, they may trigger a stress response. A cell may delay or exit the cell cycle and enter into G0 upon expression of cyclin-dependent kinase inhibitors. At higher levels, ROS directly react with such inhibitors, leading to DNA and mitochondrial damage, oxidation of amino acids in nearby proteins, lipid peroxidation, and inactivation of specific enzymes, often resulting in apoptosis (Laloi and Havaux, 2015; Mullineaux et al., 2018).

### Parameters for Assessing Phototoxicity

Phototoxicity depends on many different factors, ranging from sample type, developmental stage, localization of the fluorescent protein(s) or dye(s) and media to excitation wavelength(s), microscopy method and the precise image acquisition parameters (Laissue et al., 2017). For this study, the key parameters we consider (as shown in Table 1) are 1) the physico-optical parameters on the side of the fluorescence excitation, and 2) the biological readouts used to assess phototoxic effects. Physical parameters comprise the power of the excitation light (in mW), the intensity of the excitation light (i.e., the power per area, also known as irradiance or surface power density and in this study measured in mW/cm^2^), the time the sample is exposed to the excitation light (i.e., the exposure time) measured in seconds (or minutes or hours) and the total light dose (defined as the product of power and exposure time) measured in mJ.

**TABLE 1:** Terminology and units for the key parameters used in this study.

Physical parameters	Unit
Intensity (irradiance, surface power density)	mW/cm^2^
Exposure time (illumination time)	sec
Power	mW
Light dose (power x exposure time)	mJ
**Biological parameters**	**Unit**
Cell motility	nm/s
Duration of mitosis	min
Gene copy number (RT-qPCR)	Fold-change

### Biological Readouts of Phototoxicity: Morphology, Dynamics and Gene Expression

A crucial consideration for imaging live cells is how to assess phototoxicity in a given sample. Different approaches have been used and greatly vary in their sensitivity. Readouts range from viability (live/dead cells) and cellular morphology on the rather blunt end of the assessment criteria spectrum to the dynamics of a biological process and gene expression on the sensitive readout end.

In this study, we use a sensitive and straight-forward method to establish a dose-response curve for phototoxicity in mammalian cell line models. Specifically, we assess the effect of blue light, as commonly used for GFP and YFP excitation, on a motile mammalian cell line. Phototoxic effects are measured using a sensitive dynamic process, showing that motility can be used to reliably assess subtle positive and negative effects of illumination. For highly sensitive detection of the effect of blue light, we analysed the expression of genes involved in oxidative stress.

## Results

### Blue Light has Intensity-Dependent Positive and Negative Effects on PC3 Motility

**FIGURE 2:**
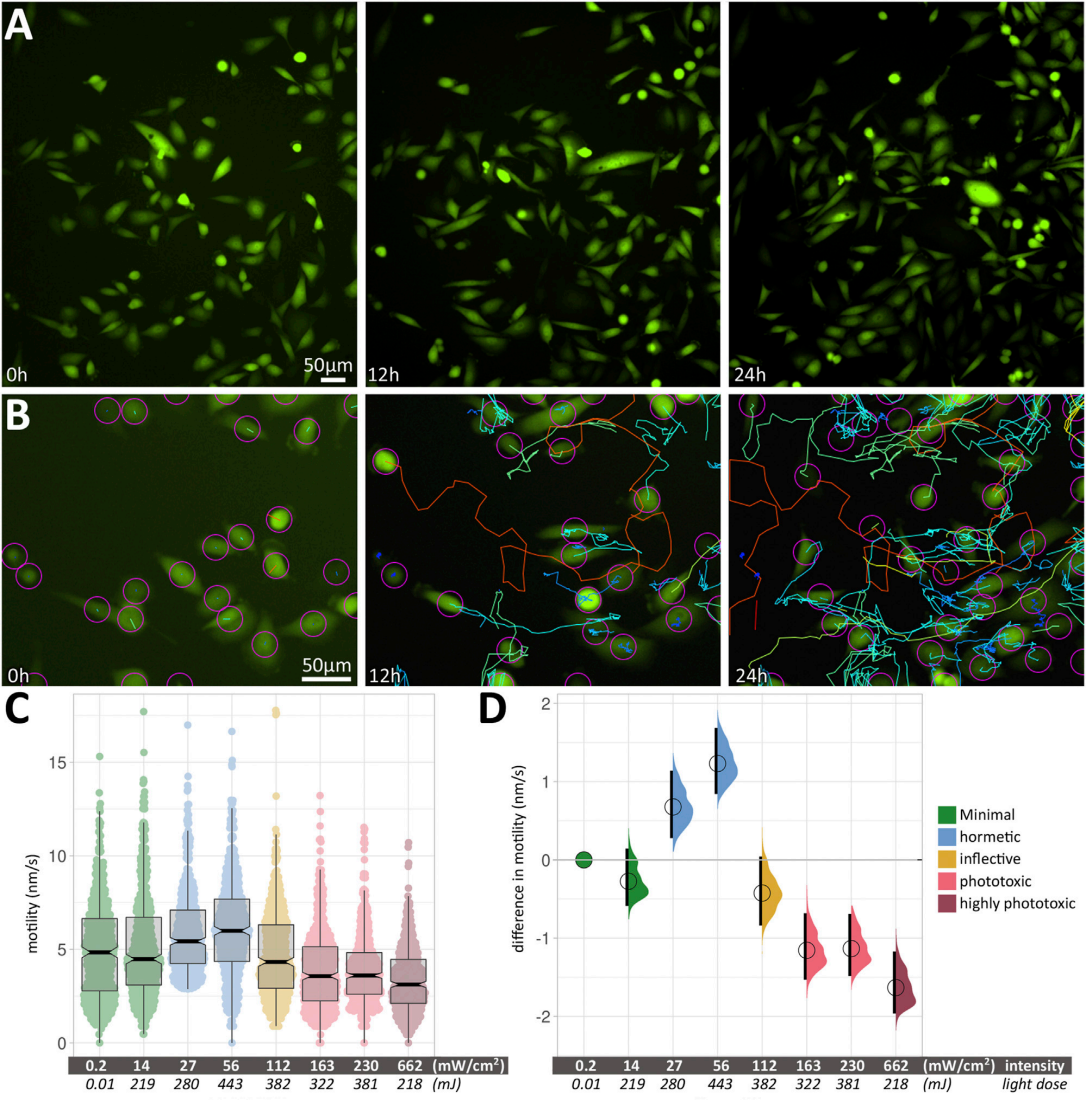
**(A)** Large field-of-view of fluorescent PC3 cells from a 24 h time-lapse movie. Three images from timepoints 0, 12 and 24 h change the appearance due to the displacement of motile cells. **(B)** An example of the automated cell tracking used to determine cell speeds. As in **(A)**, three images from timepoints 0, 12 and 24 h are shown, along with colour-coded tracks. Blue and green tracks show low speeds around 1–5 nm/s, while orange to red tracks shows fast-moving cells at around 12–15 nm/s. **(C)** Box plots of cell motility at different intensities. The *Y* axis shows cell motility, measured in nm/s. Within each box (interquartile range), the thick black line shows the median speed for each condition, and indentations depict its 95% compatibility interval. The data points used to produce the box plots are overlaid in colour. **(D)** Relative differences in motility between conditions are shown as relative effect sizes. Data points and effect sizes share the same colour-coding depicted in the legend inset in Figure 2C. The difference between median values is determined relative to the green minimal-intensity conditions and indicated with a circle. The compatibility interval is derived from the bootstrap distribution and indicated with the black vertical bars. See Supplementary Table S1 for 95% CI and *p*-values.

We acquired images of motile, fluorescent PC3-GFP cells (the prostate cancer cell line PC3, stably transfected with a GFP expression vector) in large fields-of-view (FOVs) over 24 h (Figure 2A) at different excitation light intensities and exposure times. Within a given condition, cell motility varied considerably between measured cells; an example is shown in Figure 2B, with median speeds ranging from 0.67 nm/s (blue tracks) to 24.12 nm/s (red tracks). The median speeds of 500 cells were measured for each experimental condition. Figure 2C shows the box plots of cell motility at different intensities. Thick black lines show the median speed for each condition, and indentations in the box depict its 95% compatibility interval (CI). Green signifies minimal intensity conditions, where 0.2 mW/cm^2^ was obtained using brightfield illumination without fluorescence excitation, providing a least-invasive base line speed. 14 mW/cm^2^ was used for minimally invasive fluorescence excitation with blue light (480 ± 30 nm). This intensity was doubled twice (27 and 56 mW/cm^2^, respectively), leading to increased cell motility (blue). Further doubling (112 mW/cm^2^) reduces the cell speed again (yellow) to a level close to the minimally invasive speed. Increasing intensities further (163, 230 and 662 mW/cm^2^) lead to a significant reduction in median cell speeds (light red/dark red).

The corresponding relative differences in motility between conditions are shown in Figure 2D as relative effect sizes (Goedhart, 2019) and clearly show positive and negative effects of blue light excitation on PC3-GFP cell motility. Data points and effect sizes share the same colour-coding depicted in the legend inset in Figure 2C Experimental conditions and statistical parameters are summarised in Supplementary Table S1.

### Intensity, Not Total Light Dose, Determines the Effect of Blue Light on Cell Motility

A pivotal finding was that the effect of blue light on cell motility scales with intensity (mW/cm^2^), not total light dose (mJ). Grouping median speeds according to intensity showed a clear biphasic response (Figures 2C,D), with an initial positive effect (increased cell motility) followed by a negative one (reduced cell motility). Conversely, total light dose cannot be used to explain the observed patterns, as nearly identical light doses of 219 and 218 mJ had drastically different effects on cell motility. A similar difference in response can be seen at nearly identical light doses of 382 and 381 mJ, where the lower intensity has a hormetic effect, while the higher intensity leads to a significantly reduced speed.

We used non-fluorescent brightfield microscopy (with differential interference contrast (DIC) for optical contrast). Since no fluorescence, and hence no blue light illumination, was used in this condition, it provided a non-invasive baseline for all following experiments. For the first fluorescence condition, we used very low intensity (14 mW/cm^2^). Long exposure times were needed to collect sufficient light for acceptable image contrast. This condition led to a slight, but non-significant decrease in cell motility compared to the non-invasive baseline. Doubling that initial low blue light intensity twice (i.e., 27 and 56 mW/cm^2^, respectively) led to a significant increase in cell motility. This positive effect was surprising, since hormesis has not been attributed to the short wavelengths used here (465–495 nm).

### The Intensity-Dependent Effect on Motility Increases With the Duration of the Observation

**FIGURE 3:**
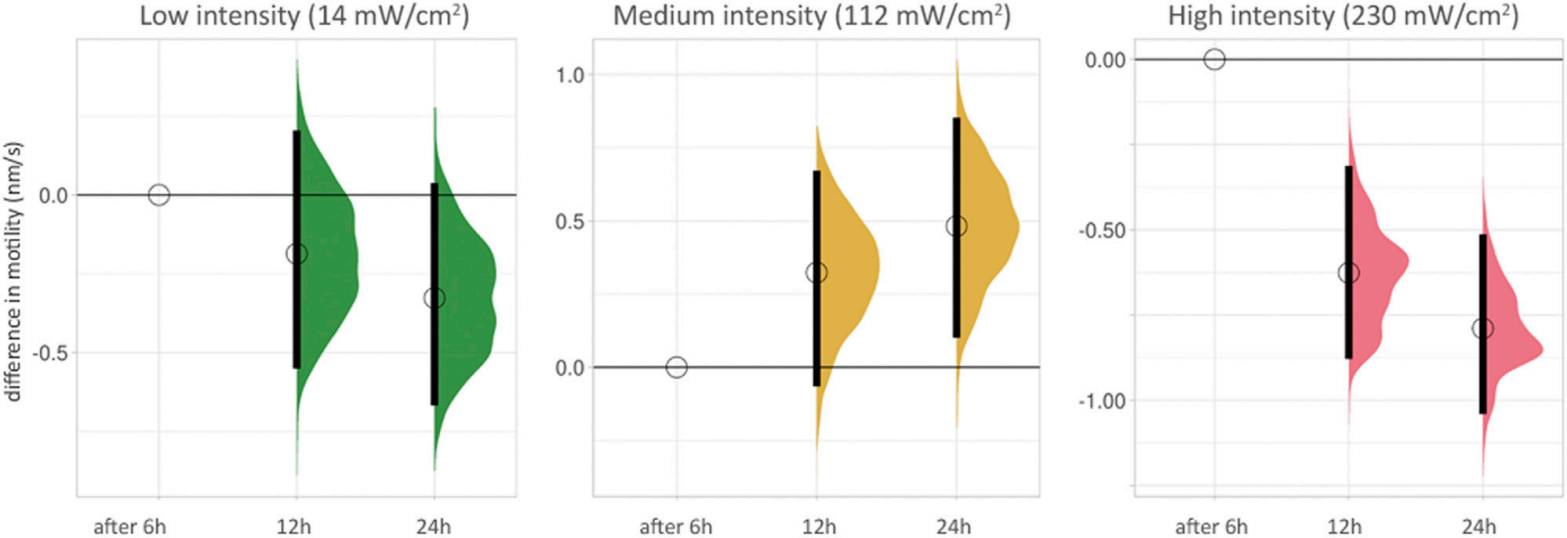
Cell motility after increasing duration of time-lapse recordings for low, medium and high intensity blue light illumination. The lowest duration (6 h) is used as baseline for each condition. The difference between median values is determined relative to the 6 h value for each condition and indicated with a circle. 95% compatibility intervals are indicated by black vertical bars. The differences between 6, 12 and 24 h at low intensity (14 mW/cm^2^, green, left side) are not significant (see also Supplementary Table S2). At medium intensity (112 mW/cm^2^, yellow, middle), a small hormetic trend appears with increasing exposure time. The two significant reductions in motility (after 12 and 24 h) are seen at high intensity (230 mW/cm^2^, red, right side).

We grouped the effect of blue light on cell motility scales according to the duration of the time-lapse recording of PC3-GFP cells with blue light at different intensities (Figure 3, Supplementary Table S2). The differences between 6, 12 and 24 h at low intensity (14 mW/cm^2^) were not significant. At medium intensity (112 mW/cm^2^), a small hormetic trend was visible with increasing exposure time. The two significant reductions in motility (after 12 and 24 h) were seen at high intensity (230 mW/cm^2^).

### Mitotic Delay Does Not Robustly Identify Phototoxic Effects

**FIGURE 4:**
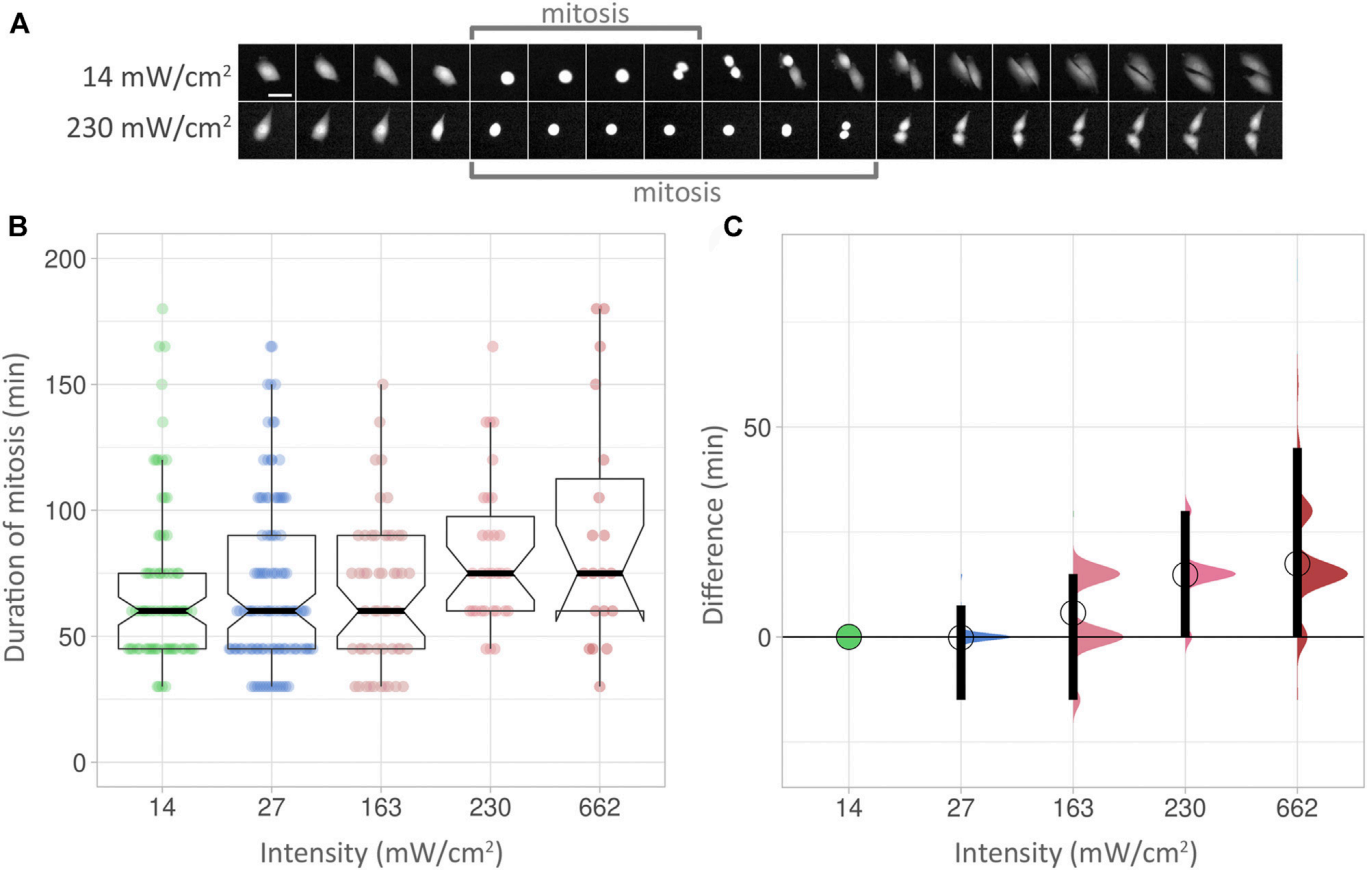
(A) Top row: Duration of mitosis examples. Images were taken at 15 min intervals. Scalebar 50 µm. **(B)** Box plots showing the duration of mitoses at different intensities over 24 h. The *Y* axis shows the duration of mitoses, measured in minutes. Within each box (interquartile range), the thick black line shows the median speed for each condition, and indentations depict its 95% compatibility interval (CI). The data points used to produce the box plots are overlaid in colour. **(C)** Effect sizes of the conditions in **(B)**. The lowest intensity (14 mW/cm^2^) is used as baseline. The differences between median values are indicated with a circle. 95% compatibility intervals are indicated by black vertical bars.

We next used mitotic delay as a biological readout to check for phototoxicity (Figure 4, Supplementary Table S3). The duration of mitoses was measured in multiple cells from start (the rounding of a cell) to end (cytokinesis of the two newly formed cells), with an image taken every 15 min for 24 h. Examples at low and high intensities are shown in Figure 4A. At low intensity (14 mW/cm^2^), the PC3 cell shown here took 60 min from rounding up to cytokinesis (indicated by “mitosis” bracket). At high intensity (230 mW/cm^2^), the process took 115 min in the cell shown. However, the statistical analysis used did not detect a significant increase in mitotic delay at higher intensities, as boxplots reveal (Figure 4B). Using effect sizes, the differences between the low intensity baseline Figure 4C, green circle and horizontal line) and subsequent median values (circles) show an increase with higher intensities. However, the 95% compatibility intervals (CI) (Figure 4C, black vertical bars) never rise above the low intensity baseline. Corresponding *p*-values are 0.299 or higher (Supplementary Table S3). Consequently, it can be neither concluded nor excluded that the higher intensities used here lead to mitotic delay.

### PC3 Cell Motility Does Not Show an Effect of Short-Term Blue Light Illumination at Moderate Intensity

**FIGURE 5:**
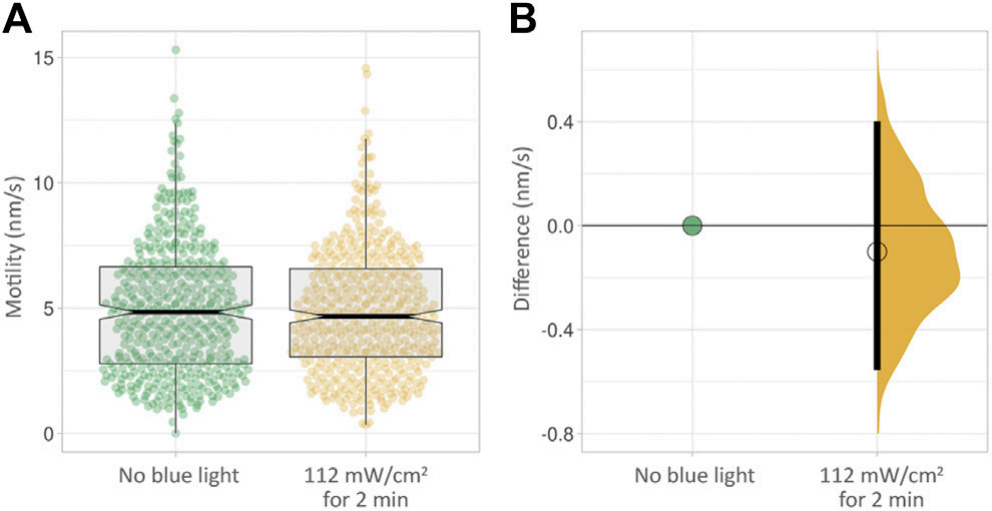
**(A)** Box plots of PC3 cell motility over 24 h with no blue light exposure (green) and after 2 min at a moderate intensity of 112 mW/cm^2^. **(B)** The effect size corresponding to **(A)**. No clear difference in motility is visible.

We next wanted to find out if short, continuous exposure to blue light at moderate intensity would result in an effect on cell motility. We subjected cells to 112 mW/cm^2^ blue light intensity, continuously for 2 minutes. This had no measurable effect on cell motility in PC3-GFP cells measured non-invasively over the following 24 h (Figure 5, Supplementary Table S4).

### Gene Expression Shows a Clear Effect of Short-Term Blue Light Illumination at Moderate Intensity

**FIGURE 6:**
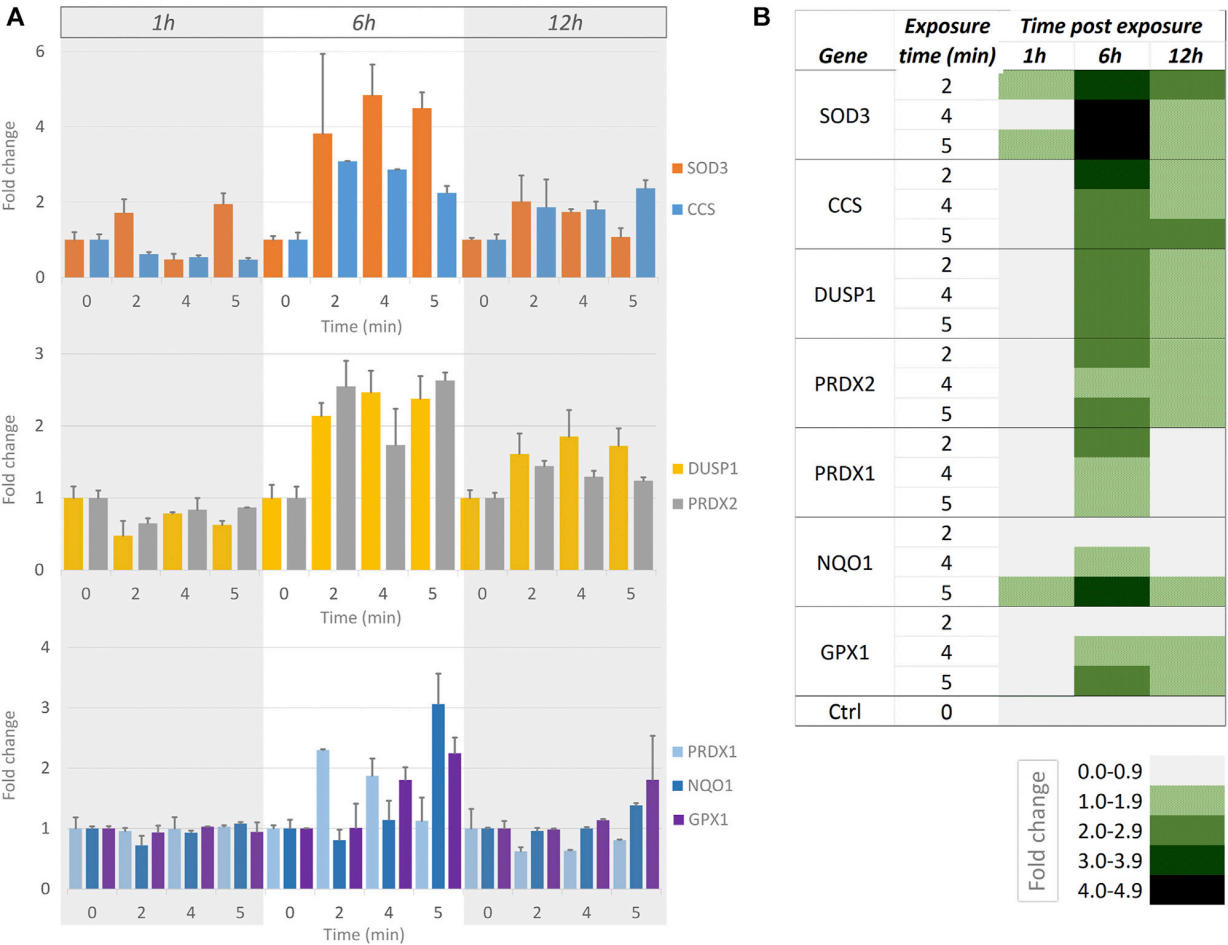
Measurements of RT-qPCR-based gene expression at different times after short exposures to moderate-intensity blue light. **(A)** Bar graphs showing gene expression fold-changes at 1, 6 and 12 h (indicated by grey/white/grey columns underlying all bar graphs) after no illumination (0) and 2, 4 and 5 min continuous illumination with blue light at 112 mW/cm^2^intensity. Top graph: SOD3 (orange) and CCS (blue). Middle graph: DUSP1 (yellow) and PRDX2 (grey). Bottom graph: PRDX1 (light blue), NQO1 (dark blue) and GPX1 (purple). Error bars indicate standard error in all graphs. **(B)** Heat map summarily visualising the responses [as shown in **(A)**]. The fold changes are indicated at the bottom of the heat map, with higher fold changes represented by darker shades of green.

We wanted to examine whether short exposures to a moderate intensity of blue light (as used in Figure 5, Supplementary Table S4) were measurable using a more sensitive readout compared to cell motility. We used RT-qPCR to determine the effect of blue light illumination upon the transcription of a panel of genes known to be important in ROS signalling (SAB target list H384 (Leone et al., 2017)). Since the generation of H_2_O_2_ is a direct result of blue light illumination [see above, Figure 1 (Alghamdi, 2017)], we chose genes that are important in the antioxidant response: *SOD3, CCS, DUSP1, PRDX1, PRDX2, NQO1*, and *GPX1*. These genes, their roles and the reference genes used are detailed in Supplementary Table S5. In these genes, a time- and dose-dependent response to illumination was evident (Figure 6). At the 1 h time point, SOD3 was the main up-regulated gene in response to illumination. At 6 h, all candidate genes were found to be up-regulated. In decreasing order of fold-changes, the expression of SOD3, CCS, DUSP1, PRDX2, PRDX1, NQO1, and GPX1 increased with higher light intensity. The transcription levels of all genes were subsequently lower 12 h post illumination.

## Discussion

### Phototoxicity Scales With Intensity, Not Light Dose

Two different approaches have commonly been used to reduce the phototoxic effects of fluorescence excitation. The first consists of lowering exposure times and increasing excitation intensity (Swedlow et al., 2009; Ettinger and Wittmann, 2014; Douthwright and Sluder, 2016). The second, converse approach favours increasing exposure times while lowering excitation intensity (Dixit and Cyr, 2003; Magidson and Khodjakov, 2013; Icha et al., 2017; Kiepas et al., 2020). Our data presented here clearly agree with the latter approach.

### Different Intensities of Blue Light Cause Hormetic or Negative Effects on PC3-GFP Cell Motility

Blue light is known to reduce motility in single cells (Mubaid and Brown, 2017; Kiepas et al., 2020). Knoll and coworkers showed that excitation at 540–585 nm, at a low intensity of 1.25 mW/cm^2^ over 60 s (resulting in 75 mJ/cm^2^), leads to rapid cytoskeletal force relaxation (Knoll et al., 2015). On the basis of these findings, we expected the blue light used in this study to have a purely negative effect on cell motility, leading to a decrease in their median speed. Here we show that the effect of blue light (480 ± 30 nm) on PC3 cell motility is biphasic (Figure 2, Table 2). Compared to a no-effect level at low intensity (14 mW/cm^2^), an increase in intensity (27 and 56 mW/cm^2^) leads to increased cell motility. An inflection point was found at moderate intensity (112 mW/cm^2^), after which higher intensities (163, 230 and 662 mW/cm^2^) cause a significant reduction in motility.

**TABLE 2:** Primers for RT-qPCR used in this study. RG: reference gene.

Gene symbol	Oligo sequence forward/Reverse	
PRDX1	TTG​CGC​GTT​TTG​TTC​TTC​CC	GCT​GTG​GCT​TTG​AAA​TTG​GG
CCS	AAC​AAC​TGC​AAC​AGC​TGT​GG	AGC​ATC​AGC​ATG​GAC​ATT​GC
DUSP1	AAC​GTC​TCA​GCC​AAT​TGT​CC	TGA​AGT​CAA​TCG​CCT​CGT​TG
GPX1	ACG​ATG​TTG​CCT​GGA​ACT​TC	ATG​TCA​ATG​GTC​TGG​AAG​CG
NQO1	TTC​CAG​AGT​AAG​AAG​GCA​GTG​C	TGG​AAG​CCA​CAG​AAA​TGC​AG
PRDX2	TTG​ATG​GCG​CCT​TCA​AAG​AG	TGG​GGC​ACA​CAA​AAG​TGA​AG
SOD3	TCT​CAC​CTT​CGC​CTT​TGT​TG	TAC​AAA​TGG​AGG​CCT​TCA​GAC​C
GAPDH (RG)	ATT​CCA​CCC​ATG​GCA​AGT​TC	ATC​GCC​CCA​CTT​GAT​TTT​GG
HPRT1 (RG)	AAC​GTC​TTG​CTC​GAG​ATG​TG	AAT​CCA​GCA​GGT​CAG​CAA​AG
TUBA1A (RG)	TGC​AAA​CAG​TCT​ACG​GAT​GC	TGC​CAA​AGA​CCA​CAT​GCT​TG
PPIA (RG)	TGC​TGG​ACC​CAA​CAC​AAA​TG	TGC​CAA​AGA​CCA​CAT​GCT​TG
TBP (RG)	CCA​CTC​ACA​GAC​TCT​CAC​AAC	CTG​CGG​TAC​AAT​CCC​AGA​ACT

Hormetic effects have been described for longer wavelengths, e.g., in the case of low-level laser therapy in the 600–700 nm range (AlGhamdi et al., 2015), but not for the shorter wavelengths used here. Longer wavelengths in the visible range have consistently been shown to be less damaging than shorter blue ones (Schneckenburger et al., 2012; Wäldchen et al., 2015; Douthwright and Sluder, 2016; Icha et al., 2017; Kilian et al., 2018). However, blue light (which we here define as ranging from around 440–500 nm) is still widely used in fluorescence microscopy, and it is unrealistic to expect that, simply due to their potentially damaging effect, excitation wavelengths below 500 nm will be avoided in the future. Many GFP-derived labels exist and are being routinely used (Remington, 2011; Rodriguez et al., 2017), so it is important to understand the effect that blue light illumination can have on mammalian cell lines (Carlton et al., 2010; Wäldchen et al., 2015; Douthwright and Sluder, 2016; Icha et al., 2017; Laissue et al., 2017).

### Mitotic Delay Is a Sparser and Less Sensitive Readout Compared to Motility

The timing of mitosis has been proposed as an ideal measure of imaging-related stress on cells (Cole, 2014). However, for the experimental setup presented here, it is a less reliable readout compared to cellular motility. While measurement of the latter was able to pick up subtle differences caused by small variations in intensity, these were missed using the timing of mitosis as a biological readout. The data are too dispersed to allow robust conclusions. More mitoses would need to be measured to decrease the uncertainty of the effect size in these examples. However, in our motile PC3 cells, movement can be assessed for the majority of cells within the field of view, whereas mitoses occur far less frequently; using the same number of fields of view, a total of 2,477 motility tracks were identified, compared to 280 mitoses. The frequency of a biological readout used to assess phototoxicity is hence an important consideration.

### A Working Model for Phototoxicity

**FIGURE 7:**
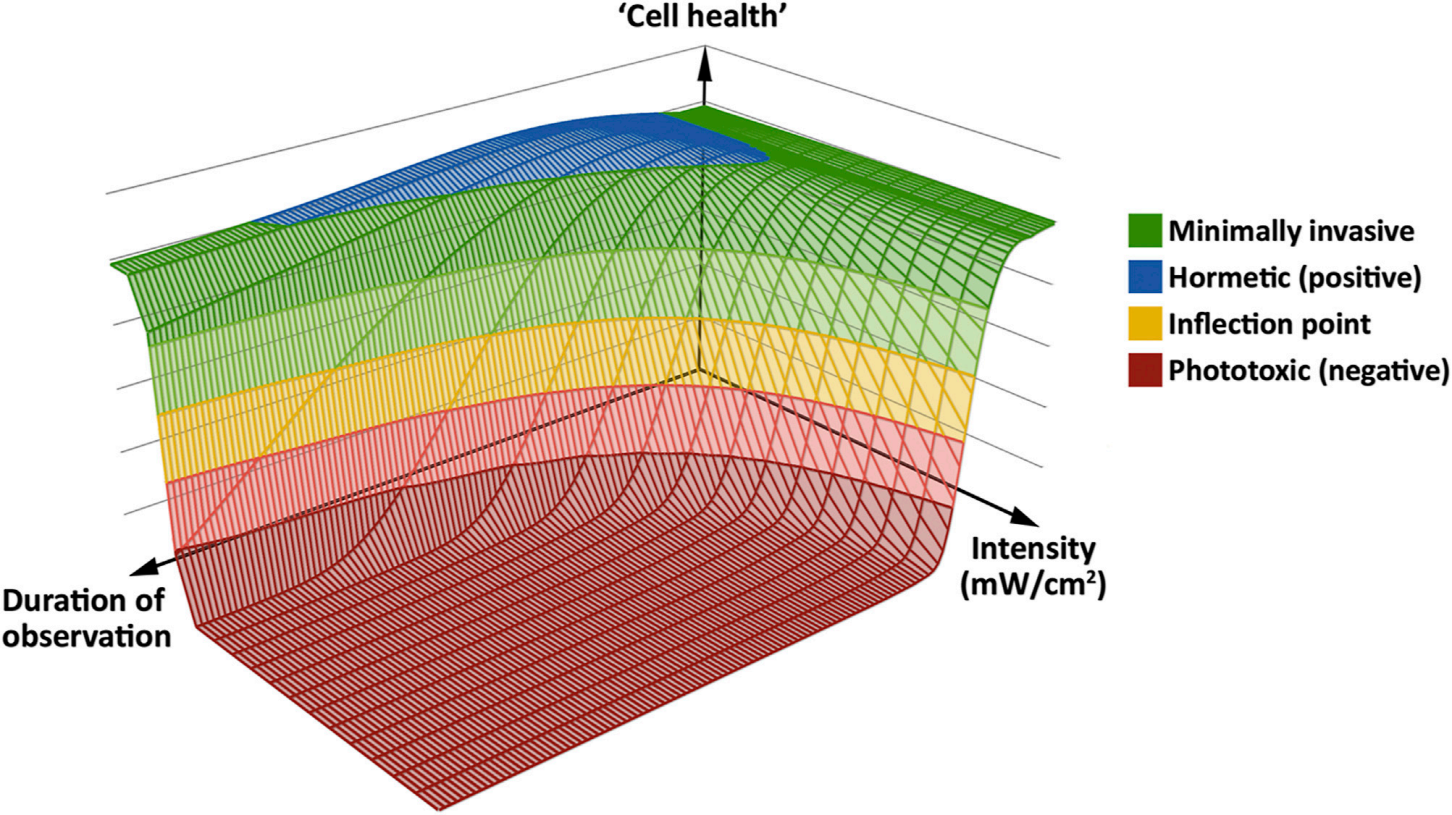
A “phototoxicity landscape” as a working model, based on the three key parameters of excitation light intensity, duration of observation and “cell health.” At low intensity, the effects of excitation light can be minimal (green) to positive (blue), with no negative effect on “cell health.” However, if excitation at low intensity occurs over a long period of observation, negative effects may start to manifest. At higher intensities, negative effects take hold more quickly. At the inflection point (yellow band), phototoxicity can reach an irreversible level, which leads to immediate or delayed cell death (red floor). Adapted from (Alghamdi, 2017).

To find an acceptable imaging mode for a given sample, ensuring that valid conclusions are drawn from its live observation using fluorescence microscopy, we believe it is helpful to have a working model in mind. Based on our findings and previous studies (Carlton et al., 2010; Dixit and Cyr, 2003; Icha et al., 2017; Kiepas et al., 2020; Laissue et al., 2017; Tinevez et al., 2012), we propose a general, simplified model for phototoxicity using a “photodamage landscape” consisting of three axes: Excitation light intensity, exposure time and cellular health. The latter is defined by the minimal or absent perturbation of the sample based on the biological readout used to assess phototoxic effects (Figure 7). Note that we here use “the cell” in a *pars pro toto* sense, i.e., as a term for any living sample, be that single-celled or multicellular. In our model, fluorescence excitation light induces the production of ROS in the observed living cell. At low intensities and short observation periods, this can be dealt with by the cell using its native ROS scavenging abilities. With moderate stress, there is also an adaptive range, entailing upregulation of genes to deal with the oxidative stress. Phototoxicity occurs, primarily through oxidative stress, when ROS are not scavenged quickly enough: The rate of repair of damaged cell components fails to keep pace with the rate of damage. If this situation persists, the cell progresses past a hypothetical inflection point, beyond which irreversible damage occurs. This results in an impairment of numerous cellular functions, a concomitant loss of physiological competence, and eventual cell death. For this progression from a physiological or adaptive state to a pathophysiological one, we propose a bi-phasic, sigmoidal effect of phototoxicity that scales with illumination intensity—similar to the model proposed for high-light responses in plants and algae (Laloi and Havaux, 2015; Mullineaux et al., 2018). The biphasic nature of phototoxicity has been described in previous studies (Dixit and Cyr, 2003; Carlton et al., 2010; Schneckenburger et al., 2012; Tinevez et al., 2012). On a molecular level, it may be related to the supralinear photobleaching of fluorescent proteins at high intensity (Cranfill et al., 2016).

The model assumes that many other factors influencing phototoxicity are already set: excitation wavelength, fluorophores (both their concentration and their subcellular localization), dark intervals between images, oxygen concentration, media, sample preparation, developmental (or cell cycle) stage, cell type and age, and synergistic effects of experimental perturbations all can affect a live sample under fluorescence microscopy observation. However, if these parameters are set, we expect any sample to qualitatively behave in a similar way.

### Gauging Phototoxicity: How Low Should It Be in a Given Experiment?

In an ideal experiment using live fluorescence microscopy, the excitation illumination should have no measurable effect on the observed sample [i.e., the no-effect level (NOEL) (Schmidt et al., 2020)]. This will however depend on how phototoxicity is assessed. Readouts such as the ratio of living to dead cells (assessed right at the end of the experiment), or using purely morphological indicators such as blebbing, are not sensitive readouts and their use is increasingly discouraged (Icha et al., 2017). A biological process will provide a more sensitive readout, such as the timing of mitosis (Cole, 2014). Its use in this study however showed limited sensitivity, and cell motility proved a better readout for PC3 cells. Increasing sensitivity further, our RT-qPCR results show that even when no differences in cell motility are found, blue light illumination may have altered gene expression. RT-qPCR thus served as a relatively simple, early ROS-induced marker at transcriptional level, revealing an effect of blue light not measurable using morphological or dynamical readouts. Note however that verifying whether the upregulated transcription of mRNAs translates into corresponding mature and active proteins cannot be answered by RT-qPCR.

This leads to two conclusions: 1) Finding sensitive methods to quantify the effect of excitation light is key. To encourage the usage of such methods and lower the effort threshold for such experiments to be included in live imaging studies using fluorescence microscopy, it is also important that they be as simple, as robust and as widely applicable as possible. 2) While it is possible that in certain experimental setups, phototoxicity can be entirely avoided, this is unlikely to apply to many experiments where higher light intensities are used to achieve the requisite spatial and/or temporal resolution of the biological process of interest. However, it is in any case fundamentally important to identify how much the applied illumination affects the sample—and biological process—in question. In all cases, illumination should be lowered to produce the minimal amount of image contrast that allows quantification of the acquired datasets.

In practical terms, this means that two fundamental questions need to be addressed prior to acquiring images for a series of experiments based on fluorescence microscopy. We propose the following steps:

1. What is the temporal and spatial resolution you require to observe the biological process of interest?
2. What is the minimal contrast required to quantify this? Consult existing literature to find initial values, then run tests to determine a narrow range for these fundamental parameters.
3. Choosing the most sensitive biological readout possible, establish a dose-response curve: At first, an intensity that produces obvious signs of phototoxicity can be used to determine the damaging end of the excitation intensity range. Now continuously reduce the excitation intensity until arriving at a minimally (or ideally non-) invasive level while still acquiring images with sufficient contrast for quantification. A reduced excitation intensity has the advantages of reducing phototoxicity and photobleaching, and may allow extending the duration of observation.

## Materials and Methods

### PC3 Cell Preparation and Image Acquisition

We used the human prostate cancer cell line PC3 (ATCC CRL-1435, Manassas, VA), stably transfected with an empty GFP plasmid for cytoplasmic expression of GFP. The cells were grown in RPMI-1640, supplemented with 10% FBS, 1% penicillin/streptomycin and 1% l-glutamine. 80–90% monolayer confluence was reached 24 h before imaging. Cells were detached from the flask with Tryp LE™, and 2 × 10^4^ cells were seeded in 96 microwell plates (ibidi GmbH, Munich, Germany). Samples were imaged at 37°C and 5% CO_2_ in a stage-top incubator (Model H301, OkoLab, Italy), with each imaged well surrounded by empty wells to avoid “splash damage” of light. Multi-location time lapse images were taken using widefield fluorescence microscopy. A Nikon Eclipse Ti-E main body was used with an automated stage for multipoint acquisition and NIS-Elements (version 3.21.03, build 705 LO) for control. Microscope objectives were a Nikon CFI PlanFluor 10x (NA 0.3, WD 16 mm) or a Nikon S Fluor 20x (NA 0.75, WD 1 mm). The light source was a metal halide Nikon Intensilight. GFP was excited with a single band emission filter at 480 ± 15 nm and its emission collected with hard-coated interference filters, using a chromatic reflector at 505 nm and a single band emission filter at 535 ± 20 nm (Chroma Technology Corp., VT, United States). Differential interference contrast (DIC) images for cellular outlines were acquired using an incandescent tungsten white light source. Two-dimensional time-lapse series were acquired with an Andor camera (Luca-R DL-626, Andor Technology, United Kingdom), with an image being taken every 15 min for at least 24 h. Intensity (irradiance) was estimated using the power (measured with an ML9002A optical handy power meter (Anritsu Corp., Japan) at sample height) and the corresponding field of view’s diagonal to calculate the circular area of illumination.

### Image Analysis

Image analysis was done using Fiji v1.53c (Schindelin et al., 2012) with the TrackMate plugin (Tinevez et al., 2017). A blob diameter of 28′000 nm and noise threshold of 0.8 were used with a Laplacian of Gaussian detector using sub-pixel localisation accuracy. Next, we selected the Linear Assignment Problem (LAP) tracker using frame-to-frame linking of 15’000 nm, track segment gap closing of 40’000 nm with a maximum frame gap of 2, and finally a track segment splitting of 15,000 nm. Median velocity was used as the measure of cell motility.

Between 56 and 131 tracks per single well in a 96-well plate were measured (average of 88 ± 22 tracks per well). We found that at a low count (60 tracks), a clear difference (1.05 nm/s) was found between two randomly chosen wells acquired in identical imaging conditions. Increasing the number of tracks to 240 by determining the median speeds in three wells, this difference was roughly halved (0.59 nm/s). Doubling the number of imaged wells to six (increasing the readout to 480 tracks) resulted in almost identical values for median speeds (difference 0.12 nm/s). We settled on the measurement of 500 tracks for each imaging condition to ensure a reliable readout of median cell speeds.

Mitotic delay analysis was conducted by observing the number of frames each cell took to complete mitosis (Figure 4A). Frames were taken in 15-min intervals. The starting point was a rounded cell; the end point was arrived at once the mitotic cell had split it into two separate cells.

### RNA Extraction and Quantitative Real-Time PCR

Cells for quantitative real-time PCR (RT-qPCR) were seeded on three 12 well plates. Each plate was exposed briefly (2, 4 and 5 min) to moderate-intensity blue light (112 mW/cm^2^). Controls were not exposed to blue light. After blue light exposure, RNA extraction was carried out on separate multiwell plates after 1, 6 and 24 h, respectively. All RNA samples from each well were collected in separate Eppendorf tubes and stored at - 80°C. Total RNA was extracted from PC3 cells using CellAmp Direct RNA Prep kit for RT-qPCR and Protein Analysis Kit (Takara Bio Inc., Japan). RNA (1 µg) was treated with Ambion RNase-free DNase1 (Thermo Fisher Scientific, Waltham, Massachusetts, United States). The cDNA samples were synthesized using random nonamer primers and the First-Strand Synthesis System (Sigma-Aldrich, United States). Quantitative real-time PCR of the cDNA was performed using an EvaGreen fluorescence-based procedure with reagents purchased from Applied Biological Materials (Richmond, Canada). The primers used in this study for RT-qPCR are given in Table 2.

Relative and normalised fold expression values were calculated using the CFX Manager Software 3.1 (Bio-Rad, California, United States). A set of reference genes, glyceraldehyde-3-phosphate dehydrogenase (GAPDH), hypoxanthine guanine phosphoribosyl transferase (HPRT1A) peptidylprolyl isomerase (PPIA) and TATA box binding protein (TBP) were checked with the population of cDNA samples. The entire Ct dataset was analysed using qBASE+ (Biogazelle) implemented in CFX Manager Software 3.1 (Bio-Rad). The reference genes that showed higher expression stability were PPIA/TBP (CV = 0.20; M = 0.56) followed by HPRT1 (CV = 0.56; M = 0.94), TUB1A (CV = 0.66; M = 1.08) and GAPDH (CV = 0.72; M = 01.20) for the normalization purpose (Vandesompele et al., 2002). The expression stability values calculated for the pairs of reference genes PPIA/TBP are inside the ranges proposed by Hellemans and co-workers (Hellemans et al., 2007) as acceptable for heterogeneous (M ≤ 1; CV ≤ 0.5) and relatively homogeneous (M ≤ 0.5 and CV ≤ 0.25) sample panels. We consider a relative quantification (RQ) significant when there is a minimum two-fold change: RQ of more than 2 or less than 0.5.

### Statistical Analysis With PlotsOfDifferences

Data analysis was done using PlotsOfDifferences (Goedhart, 2019). Data were visualised using box plots (with 95% compatibility intervals indicated by indentations) and a quasi-random distribution of data, along with displaying effect sizes. Corresponding *p*-values were produced using randomisation tests (Hooton, 1991; Nuzzo, 2017; Goedhart 2019).

## Conclusion

Our study shows that 1) even very low intensity alters the experimental outcome in the case of PC3-GFP motility illuminated by blue light, 2) effects can be positive or negative, 3) effects scale with intensity, not light dose, and 4) changes in gene expression may long precede morphological or cell dynamical parameters. All of which underlines the crucial importance of assessing phototoxicity in live imaging studies to avoid drawing misleading conclusions.

## Data Availability

The raw data supporting the conclusion of this article will be made available by the authors, without undue reservation.
