# Supplementary material for: Assessing Phototoxicity in a Mammalian Cell Line: How Low Levels of Blue Light Affect Motility in PC3 Cells
Source: Front Cell Dev Biol. 2021 Dec 17;9:738786. doi: 10.3389/fcell.2021.738786 (PMC8718804; doi:10.3389/fcell.2021.738786)
Supplement: Supplementary file 1 [file DataSheet1.PDF]

# Supplementary Material

(For Alghamdi et al., "Assessing phototoxicity in a mammalian cell line: How low levels of blue light affect motility in PC3 cells"; this issue).

## *Supplementary Tables*

***Table S1*** *Conditions, speeds and differences of 24h time-lapse recordings depicted in Fig. 2.*

| Intensity ($mW/cm^2$) | Light dose (mJ) | Median speed (nm/s) | MAD | difference (nm/s) | 95% CI median | p-value for median |
|---|---|---|---|---|---|---|
| 0.2 | 0.014 | 4.84 | 1.97 | Ref. | 4.7 - 5.19 | 1 |
| 14 | 219 | 4.48 | 1.70 | - 0.275 | 4.93 - 5.43 | 0.1 |
| 27 | 280 | 5.43 | 1.40 | 0.672 | 5.71 - 6.11 | <0.001 |
| 56 | 443 | 5.99 | 1.67 | 1.229 | 5.86 - 6.33 | <0.001 |
| 112 | 382 | 4.33 | 1.64 | - 0.427 | 4.54 - 4.97 | 0.042 |
| 163 | 322 | 3.57 | 1.35 | - 1.158 | 3.75 - 4.13 | <0.001 |
| 230 | 381 | 3.6 | 1.07 | - 1.143 | 3.74 - 4.08 | <0.001 |
| 662 | 218 | 3.12 | 1.13 | - 1.644 | 3.22 - 3.53 | <0.001 |

***Table S2*** *Experimental conditions and numerical statistics for Fig. 3.*

| **Intensity ($mW/cm^2$)** | **Duration (h)** | **n** | **95% CI median (nm/s)** | **Median (nm/s)** | **difference** | **p-value for median** |
|---|---|---|---|---|---|---|
| 14 | 0-6 | 256 | 4.48 - 5.12 | 4.78 | | 1 |
| 14 | 0-12 | 328 | 4.46 - 4.86 | 4.61 | -0.183 | 0.368 |
| 14 | 0-24 | 500 | 4.35 - 4.64 | 4.48 | -0.326 | 0.133 |
| 112 | 0-6 | 363 | 3.58 - 4.1 | 3.85 | | 1 |
| 112 | 0-12 | 500 | 3.9 - 4.4 | 4.18 | 0.324 | 0.104 |
| 112 | 0-24 | 500 | 4.05 - 4.52 | 4.33 | 0.483 | 0.018 |
| 230 | 0-6 | 258 | 4.19 - 4.55 | 4.44 | | 1 |
| 230 | 0-12 | 330 | 3.58 - 3.99 | 3.78 | -0.626 | <0.001 |
| 230 | 0-24 | 500 | 3.41 - 3.77 | 3.6 | -0.789 | <0.001 |

***Table S3*** *Mitotic delay numerical statistics for Fig. 4*

| ***Intensity ($mW/cm^2$)*** | ***n*** | ***Median (nm/s)*** | ***95% CI median (nm/s)*** | ***p value for median*** |
|---|---|---|---|---|
| *14* | *73* | *60* | *60* | *1* |
| *27* | *106* | *60* | *60 - 67.5* | *1* |

| | | | | |
|---|---|---|---|---|
| *163* | *51* | *60* | *45 - 75* | *1* |
| *230* | *31* | *75* | *60 - 90* | *0.348* |
| *662* | *19* | *75* | *60 - 105* | *0.299* |

**Table S4** *Experimental conditions and numerical statistics for Fig. 5.*

| **Condition** | **N** | **median** | **95CI median** | **difference** | **p – value (median)** |
|---|---|---|---|---|---|
| No blue light | 449 | 4.84 | 4.35 - 5 | reference | 1 |
| 112 $mW/cm^2$ blue light for 2 min | 446 | 4.67 | 4.3 - 4.94 | - 0.17 | 0.556 |

***Table S5:*** *Gene candidates used in gene expression analysis*

| **Gene (abbr.)** | **Gene name** | **Description** |
|---|---|---|
| SOD3 | superoxide dismutase 2 | • Member of the iron/manganese superoxide dismutase family<br>• mitochondrial protein product converts superoxide byproducts to hydrogen peroxide and diatomic oxygen |
| CCS | copper chaperone for superoxide dismutase | • Delivers Cu to copper/zinc superoxide dismutase<br>• may activate copper/zinc superoxide dismutase through direct insertion of the Cu cofactor |
| DUSP1 | dual specificity phosphatase 1 | • Can dephosphorylate MAP kinase MAPK1/ERK2<br>• plays an important role in the human cellular response to environmental stress & negative regulation of cellular proliferation<br>• protein product can make some solid tumors resistant to both chemotherapy and radiotherapy |
| PRDX1 & PRDX2 | Peroxiredoxins 1 and 2 | • peroxiredoxin family of antioxidant enzymes<br>• reduce hydrogen peroxide and alkyl hydroperoxides<br>• may play an antioxidant protective role in cells |
| NQO1 | NAD(P)H quinone dehydrogenase 1 | • Encodes a cytoplasmic 2-electron reductase<br>• Protein product reduces quinones to hydroquinones<br>• prevents the one electron reduction of quinones that results in the production of radical species |
| GPX1 | glutathione peroxidase 1 | • Protein product catalyzes reduction of organic hydroperoxides and hydrogen peroxide by glutathione<br>• Protects cells against oxidative damage |
| GAPDH | | Reference gene |
| HPRT1 | | Reference gene |
| TUBA1A | | Reference gene |
| PPIA | | Reference gene |
| TBP | | Reference gene |
